# Identification and Characterization of 33 *Bacillus cereus sensu lato* Isolates from Agricultural Fields from Eleven Widely Distributed Countries by Whole Genome Sequencing

**DOI:** 10.3390/microorganisms8122028

**Published:** 2020-12-18

**Authors:** Athanasios Zervas, Marie Rønne Aggerbeck, Henrietta Allaga, Mustafa Güzel, Marc Hendriks, IIona Jonuškienė, Orsolya Kedves, Ayse Kupeli, Janja Lamovšek, Pascal Mülner, Denise Munday, Şahin Namli, Hilal Samut, Ružica Tomičić, Zorica Tomičić, Filiz Yeni, Raida Zribi Zghal, Xingchen Zhao, Vincent Sanchis-Borja, Niels Bohse Hendriksen

**Affiliations:** 1 Department of Environmental Science, Aarhus University, 4000 Roskilde, Denmark; az@envs.au.dk (A.Z.); mrag@envs.au.dk (M.R.A.); 2 Department of Microbiology, University of Szeged, 6726 Szeged, Hungary; henrietta.allaga@gmail.com (H.A.); kedvesorsolya91@gmail.com (O.K.); 3 Department of Food Engineering, Hitit University, 19030 Çorum, Turkey; musguzel@gmail.com; 4 Wageningen Plant Research, Wageningen University, 6708PB Wageningen, The Netherlands; marc.hendriks@wur.nl; 5 Department of Organic Chemistry, Kaunas University of Technology, 50254 Kaunas, Lithuania; ilona.jonuskiene@ktu.lt; 6 Department of Food Engineering, Middle East Technical University, 06800 Ankara, Turkey; aysekupelii@gmail.com (A.K.); snamli@metu.edu.tr (Ş.N.); hilal.samut@metu.edu.tr (H.S.); filizyeni@gmail.com (F.Y.); 7 Plant Protection Department, Agricultural Institute of Slovenia, 1000 Ljubljana, Slovenia; Janja.Lamovsek@kis.si; 8 Graz University of Technology, 8010 Graz, Austria; pascal.muelner@tugraz.at; 9 Sumitomo Chemical Agro Europe, 1260 Nyon, Switzerland; Denise.MUNDAY@sumitomo-chem.fr; 10 Faculty of Technology, University of Novi Sad, 21000 Novi Sad, Serbia; ruzica.tomicic@yahoo.com; 11 Institute of Food Technology, University of Novi Sad, 21000 Novi Sad, Serbia; zorica.tomicic@fins.uns.ac.rs; 12 Sfax Preparatory Engineering Institute, Sfax University, P.O. Box 1172, 3000 Sfax, Tunisia; raida_zz@yahoo.fr; 13 Centre of Biotechnology of Sfax, Biopesticides Laboratory, P.O. Box 1177, 3018 Sfax, Tunisia; 14 Laboratory of Food Microbiology and Food Preservation, Ghent University, 9000 Ghent, Belgium; Xingchen.Zhao@UGent.be; 15 Micalis Institute, INRA, Université Paris-Saclay, 78 350 Jouy-en-Josas, France; vincent.sanchis-borja@inra.fr

**Keywords:** ecotypes, patho-types, phylogeny, geographical-distribution, *Bacillus mycoides*, *Bacillus toyonensis*, *Bacillus mosaicus*, *Bacillus thuringiensis*

## Abstract

The phylogeny, identification, and characterization of 33 *B. cereus sensu lato* isolates originating from 17 agricultural soils from 11 countries were analyzed on the basis of whole genome sequencing. Phylogenetic analyses revealed all isolates are divided into six groups, which follows the generally accepted phylogenetic division of *B. cereus sensu lato* isolates. Four different identification methods resulted in a variation in the identity of the isolates, as none of the isolates were identified as the same species by all four methods—only the recent identification method proposed directly reflected the phylogeny of the isolates. This points to the importance of describing the basis and method used for the identification. The presence and percent identity of the protein product of 19 genes potentially involved in pathogenicity divided the 33 isolates into groups corresponding to phylogenetic division of the isolates. This suggests that different pathotypes exist and that it is possible to differentiate between them by comparing the percent identity of proteins potentially involved in pathogenicity. This also reveals that a basic link between phylogeny and pathogenicity is likely to exist. The geographical distribution of the isolates is not random: they are distributed in relation to their division into the six phylogenetic groups, which again relates to different ecotypes with different temperature growth ranges. This means that we find it easier to analyze and understand the results obtained from the 33 *B. cereus sensu lato* isolates in a phylogenetic, patho-type and ecotype-oriented context, than in a context based on uncertain identification at the species level.

## 1. Introduction

*Bacillus cereus sensu lato*, also known as the *B. cereus* group, consists of Gram-positive, rod-shaped, spore-forming bacilli, commonly isolated from soil, and other environmental and food matrices. The founders of the group are the well-known species *B. cereus sensu stricto* (s.s.), an opportunistic pathogen and food spoiler, *B. anthracis*, the etiological agent of anthrax, and *B. thuringiensis*, an entomopathogen, with specific strains widely used as biocontrol agents. The group also encompasses species of minor economic or medical importance, such as *B. weihenstephanensis, B. mycoides* and *B. pseudomycoides*. Since 2011, another 15 species have been described as plausible members of the group, however, the taxonomic and phylogenetic relationships of these bacteria are still unclarified. During the last 20 years, genotypic methods have revealed that the traditional practical classification and identification methods are not necessarily consistent with the group’s phylogenetic classification. Notably, fluorescent amplified fragment-length polymorphism patterns (AFLP) and multiple locus sequence typing (MLST) have revealed the existence of three broad phylogenetic clades further divided into seven major groups, mentioned as phylogenetic groups I to VII [1,2,3]. These, often, do not correspond to their phenotypic identification, e.g., strains affiliated to *B. thuringiensis* are found in at least four of the phylogenetic groups since their specific insecticidal feature is plasmid-borne and can be horizontally transferred [1]. Lately, whole genome sequencing has been used for analyzing the phylogeny of the group. Liu et al. [4] analyzed 224 *B. cereus* group strains by the Genome BLAST (Basic Local Assignment Search Tool) Distance Phylogeny approach based on digital DNA:DNA hybridization. Their analysis separated the strains into 30 clusters, representing eleven known, partially merged species, and accordingly, 19–20 putative novel species. This approach has recently been used to describe a general automated high-throughput platform for state-of-the-art genome-based taxonomy [5]. Bazinet [6], who analyzed 498 *B. cereus* group genomes with different phylogenetic analysis tools, found that, irrespective of data source and analysis methodology, the three-clade and seven-group classification system was recapitulated, suggesting that the broad phylogenetic structure suggested by AFLP and MLST has been inferred correctly. Recently, Carroll et al. [7] used an average nucleotide identity (ANI) approach to make a proposal for a new taxonomic nomenclature of the *B. cereus* group by analyzing 2231 genomes, proposing 18 genomo-species to exist, of which six are undescribed. However, the analysis maintained the known overall phylogenetic structure with three clades and seven groups. In addition, they identified outlying groups—groups that still need to be characterized in detail.

Guinebretiere et al. [2] recognized an adaptation to different growth temperatures within the *B. cereus* group, concurrent with the above-mentioned seven phylogenetic groups, for example, group VI members are psychro-coldtolerant with a growth range of between 5 and 37 °C, while members of group VII are thermotolerant with a growth range between 20 and 50 °C. This adaptation has been corroborated by a strong observed relationship between divergence in ribosomal proteins, the seven phylogenetic groups and an adaptation of the *B. cereus* group to different temperature growth ranges [8]. These findings, along with other studies, suggested that the *B. cereus* group should be considered a single evolutionary unit characterized by clonal expansion and adaptation to various environmental factors that led to the formation of distinct phenotypes, also mentioned as ecotypes, within major phylogenetic lineages [9,10,11]. These suggestions relate to the fact that cytotoxic activity levels and toxin distribution vary accordingly to the phylogenetic group, implying that the group differs in food poisoning potential [12].

The pathogenicity of *B. cereus* group bacteria is, apart from anthrax and some anthrax-like diseases, caused by *B. anthracis* and a few *B. cereus* strains, notably two gastro-intestinal diseases, although several somatic diseases have also been described [13]. The two gastro-intestinal diseases are the emetic syndrome and diarrheal syndrome. The emetic syndrome is caused by the toxin cereulide, which is pre-formed in food and it is primarily observed in a distinct subgroup within the phylogenetic group III of *B. cereus* [14]. The diarrheal syndrome has notably been linked to three pore-forming enterotoxins thought to be produced in the intestine. They are two three-component toxins: the nonhemolytic enterotoxin (Nhe), the hemolytic toxin (Hbl) and the single protein cytotoxin (CytK). However, phospholipases, sphingomyelinases, hemolysins, proteinases and peptidases likely represent additional virulence factors involved in the syndrome [14]. These enterotoxins and additional virulence factors are broadly distributed among the members of the *B. cereus* group. Carroll et al. [15] developed a tool for identifying the enterotoxins and some additional virulence factors for whole genome sequenced *B. cereus* group members.

The aims of this paper are to identify and characterize 34 whole genome sequenced strains isolated from agricultural soils from eleven countries in a phylogenetic context. The analyses are based on different tools available for identification based on whole genome sequences (WGS) and are compared to an ecotype approach for characterization, including temperature growth range and presence of virulence factors.

## 2. Materials and Methods

Surface soil samples were collected from 17 fields in 11 countries spanning approximately 2100 km from north to south (Denmark to Tunisia) and 1500 km west to east (Belgium to Turkey) during January 2019. Details about the localities are given in Table 1.

The soil samples were gently mixed in sterile plastic bags. Approximately 2.5 g of the mixed soil was added to 25 mL demineralized water and further mixed for 5 min by a multi-wrist shaker (Lab-line, speed 5). Afterwards, 10 mL of the suspensions were heat-treated in a water bath (65 °C for 35 min). Ten-fold serial dilutions of the suspensions were plated on T3 sporulation agar [16] and incubated for one day at 30 °C. Two colonies having a rugose, ice-crystal-like appearance and a diameter > 1 mm were randomly selected as being *B. cereus sensu lato* and sub-cultured on T3-agar. 

For DNA preparation, bacterial cultures were plated on Luria-Bertani (LB) agar and incubated overnight at 30 °C. Bacterial biomass, corresponding to a 1–2 mm diameter colony, was transferred to 200 μL Tris-EDTA buffer. Bacteria were lysed by incubation at 102 °C for 10 min, and debris was removed by centrifugation at 15,000× *g* for 3 min. The DNA-containing supernatant was transferred to a new microcentrifuge tube and stored at 4 °C. Multiplex PCR for the affiliation of the bacteria to the *B. cereus*-group were run as described in Hansen et al. [17]. PCR of the 16S-23S rRNA gene (rDNA) spacer region with the L1-G1 [18] primer set was used as a control of DNA quality and for the procedure. PCR products were visualized by 1.5% agarose gel electrophoresis, using MW VI (Roche) as the molecular weight marker, on an Azure c200 gel imaging system (Azure Biosystems, Dublin, CA, USA). 

Thirty-four isolates for whole genome sequencing were grown on Luria Bertani broth (LB) at room temperature for 24 h. DNA was extracted using the Ultraclean Microbial DNA isolation kit (Qiagen, Hilden, Germany) and quantified on a Qubit 2.0 (Life Technologies, Carlsbad, CA, USA). Sequencing libraries were prepared using the Nextera XT sample preparation kit (Illumina, Cambridge, United Kingdom) following the manufacturer’s instructions. The libraries were sequenced on the Illumina Nextseq 500 platform using the 150 bp pair-end reads technology. The obtained raw reads were trimmed for quality, their adapters removed using fastp [19], and de novo assemblies were performed using SPAdes (version 3.14.1, St. Peterburg, Russia). Annotations of the assembled draft genomes were done using Prokka (version 1.14.5, Carlton, Australia) under default settings and databases [20].

MLST analysis on the selected strains was performed using the Center for genomic epidemiology platform for MLST [21]. This service uses a *B. cereus* MLST scheme developed by Priest et al. [1] for the analysis. The affiliation of the strains to the phylogenetic group by MLST was done using the group of its nearest neighbor, for which the affiliation is known in the MLST database.

Traditional identification was based on colony morphology, delineation to the *B. cereus* group by PCR, reaction on mannitol Egg Yolk Polymyxin agar plates, phase contrast microscopy and differentiation of *B. cereus* and *B. weihenstephanensis* by the presence of the gene for the cold-shock protein *cspA* variant specific for this species identified in the whole genome sequence [22].

Full-length 16S rRNA sequences from each sample were extracted from the resulting annotations from Prokka and aligned using MAFFT v 7.450 [23] with default settings, implemented in Geneious Prime 2020.2 (Biomatters, Auckland, New Zeland). A phylogenetic tree was constructed on RAxML (version 4.0 implemented in Geneious Prime) [24] using the GTR GAMMA model under the “Rapid Bootstrapping and search for best-scoring ML tree” algorithm with 100 bootstraps replicates, embedded in Geneious Prime 2020.2 [24].

In silico taxonomic classification of the isolates based on ANI using their draft-assembled genomes was performed using Btyper3 under default settings and adding the virulence flag to check for biovars Anthracis or Emeticus [7].

In silico taxonomic classification of the isolates based on digital DNA:DNA hybridization using their draft assembled genomes was performed using TYGS [5].

Virulence-based classification of the isolates in this analysis was performed using Btyper [15]. This tool was used for classifying isolates to phylogenetic groups based on the sequence of *panC* using the default setting corresponding to “draft genomes”, as described in the program’s manual (https://github.com/lmc297/BTyper).

Complete and draft genome assemblies analyzed in this investigation are deposited in GenBank under BioProject PRJNA681344.

## 3. Results

In this study, we isolated 34 *B. cereus* group bacteria from 17 agricultural fields originating from 11 countries (Table 1) based on colony morphology. The identity of the isolates as *Bacillus cereus sensu lato* was confirmed by a multiplex PCR assay. All the 34 isolates were whole genome sequenced. The overall genomic features of the isolates are presented in Table 2. The estimated number of bases and coding sequences (CDS) for isolate N32 is approximately two times the size of that estimated for the other 33 isolates (Table 2). Further analysis with the isolate N32 revealed that it was composed of two species related to *B. cereus sensu lato* and *Lysinibacillus* spp. For that reason, N32 is omitted from further analysis. 

The estimated number of bases and CDS vary between 5.5 and 7.1 MB and 5550 and 6433, respectively. Averages for the number of bases are 6.0 MB and 5965 for the CDS. In comparison, the number of contigs varies between 173 and 4682, with an average of 709. The number of tRNA and rRNA genes varies between 91 and 139 and 6 and 20, respectively.

Results of WGS-based MLST of the 33 isolates are presented on Table 3. The isolates comprise 29 sequence types (ST), of which 11 are new, while 11 of these sequence types are determined with some uncertainty, as no allele in the current database matches with 100% identity and coverage to the seven analyzed genes. Three of the isolates belong to sequence type 12, two belong to sequence types 56 and 434, and the remaining 26 isolates belong to different sequence types. Table 3 also highlights the six different phylogenetic groups (based on *panC* sequences) to which the isolates belong. According to Guinebretièere et al. [2], these groups are referred to as group I, II, III, IV, V and VI, to which 1, 3, 1, 10, 8 and 10 isolates belong, respectively. The division of the isolates into these groups was confirmed by the MLST analysis; however, in the case of some isolates, there is a level of uncertainty as the MLST profile is new.

**Figure 1:**
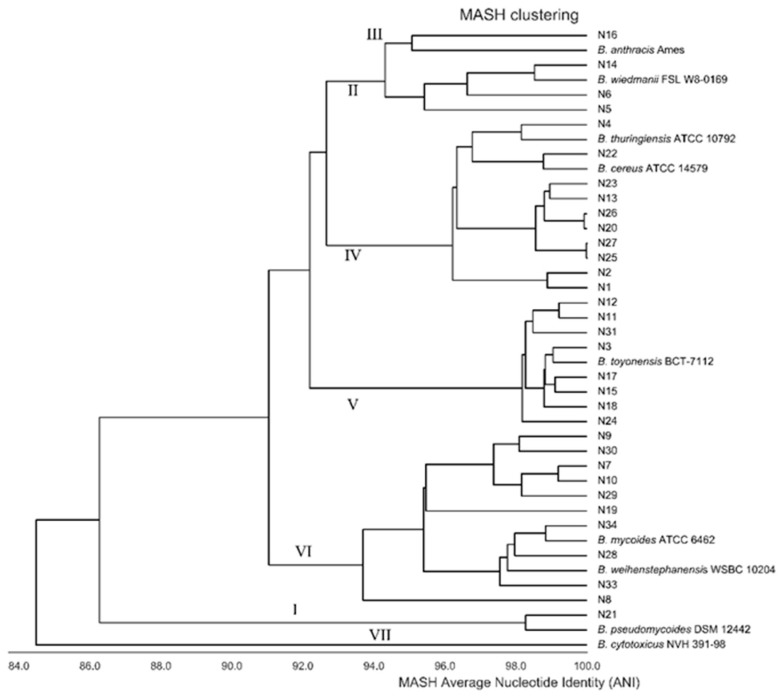
Average Nucleotide Identity (ANI)-based phylogenetic tree of 33 *B. cereus s.l*. constructed by MASH clustering. Nine strains referring to different species within the group are included. Further, the affiliation of the different clusters’ relations to the proposed phylogenetic groups I–VII [2] is shown.

Figure 1 shows a phylogenetic tree for the 33 isolates based on Average Nucleotide Identity (ANI). The phylogenetic tree indicates that this methodology divides the isolates into six major groups (I–VI). This division of the isolates between these groups corresponds exactly to the division based on *panC* sequencing and MLST.

The identification of the isolates to the species level was performed by four different methods (Table 4). Twenty-three of the isolates were identified as *B. cereus* and the rest as *B. weihenstephanensis* by traditional identification. According to 16 rRNA gene sequence analysis, twelve isolates were identified as *B. cereus*, eight as *B. mycoides*, seven as *B. wiedmanii*, two as *B. thuringiensi* and one as *B. pseudomycoides* or *B. toyonenisis*, while two were identified as *Bacillus* sp. and one isolate was not identified. The ANI-based tool in Btyper3 identified six isolates as *B. cereus SS*, four as *B. cereus* Biovar *thuringiensis*, ten as *B. mycoides*, eight as *B. toyonensis*, four as *B. mosaicus* and one as *B. pseudomycoides*. The isolates identified as *B. cereus* biovar *thuringiensis* possessed either Cry75Aa3 (N1), Vip1Ad1 and Vip2Ba2 (N2), or Vip1Ad1 and Vip2Ad1 (N25 and N27). The TYGS tool based on DNA:DNA in silico hybridization identified ten isolates as *B. toyonensis*, three as *B. mycoides*, two as *B. cereus*, two as *B. proteolyticus*, one as *B. pseudomycoides* and one as *B. wiedmanii,* while 14 isolates were identified as belonging to seven new species.

The relation between the *panC* and MLST classification of the isolates into six phylogenetic groups is shown in Table 4. The relation to the traditional identification is clear as the *B. cereus* belongs to group I, II, III, IV and V, while *B. weihenstephanensis* belongs to group VI. For the identification based on 16S rRNA genes, it is not clear, as, e.g., the isolates belonging to *B. wiedmanii* belong to groups II, IV and V, together with isolates identified as four other species, while *B. mycoides* constitute group VI, together with one *B. wiedmanii*. For the identification by the ANI-based method, the relation is clear, as the four identified species belong separately to groups I, IV, V or VI, while group II and III isolates belong to the newly proposed species *B. mosaicus*. For the DNA:DNA hybridization-based method, the relationship is unclear, except for the strains identified as *B. toyonensis*, which all except one belong to group 5. It is also worth taking into account that none of the 33 isolates belong to the same species, when identified by the four methods. 

Table 5 shows the percentage identity of nineteen translated virulence genes to the genes found in the BTyper virulence database [15]. We restricted the analysis to genes for the virulence factors and omitted regulatory elements. Further, we omitted *bceT*, as the existence of this gene is debated [25].

**Figure 2:**
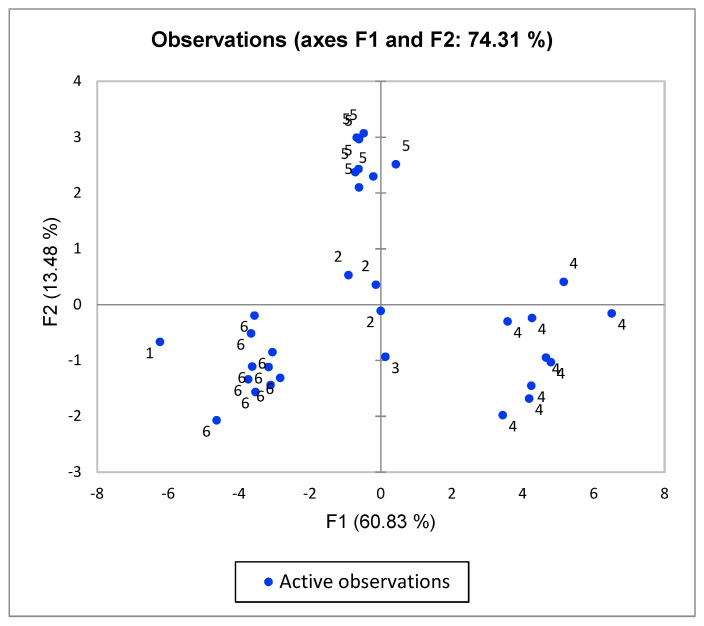
Principal component analysis (PCA) of presence and identity of 19 potential genes in 33 *B. cereuscereus sensu lato* isolates with their affiliation to six phylogenetic groups shown. F1 relates to the percentage identity of all the genes, except *sph,* and F2 relates to the percentage identity of *sph.* Each isolate (blue spots) was plotted according to its values in PC analysis.

It appears from the table that when isolates 21 (the only group I isolate) and 16 (the only group III isolate) are not taken into account, the seventeen of the nineteen virulence genes are found in almost all the isolates, except for *cytK2,* which is restricted to six of the isolates from group IV (6 out of 10) and one from group V (1 of 8), and for *hlyII,* which is present in two out of three of the isolates of group II and in eight out of ten of the isolates of group IV. Statistical analysis (Kruskal–Wallis test, *p* < 0.05, Table 5) reveals that, for all the translated genes, significant differences exist between the percent identity of at least two of the phylogenetic groups II, IV, V and VI. Group IV isolates have statistically significantly higher identity than group VI isolates for all genes except the sphingomyelinase (*sph*), while significant differences exist between group VI and groups II and V. For *sph,* significant differences exist between isolates of group II and the isolates from the other three groups. This association between the percent identity to the standard genes and the phylogenetic groups is further evidenced in a principal component analysis (PCA) performed to determine how the isolates cluster in a two-dimensional (2D) scatterplot according to phenotype combinations (Figure 2). We plotted the isolates according to their coordinates in the principal component 1 (F1) and 2 (F2). Using this coordinate system, four groups of isolates could be distinguished. It appears from the figure that F1 and F2 account for most of the initial variability in the data, with F1 describing the largest part (60.83%). The squared cosines of the variables show that the percentage identity of all the genes, except *sph,* are linked to the first axis, while *sph* is linked to the second. The PCA separates the isolates into four groups, strongly relating to their phylogenetic groups. Isolates from groups IV, V and VI comprise three clusters, with the fourth cluster comprised of the three group II isolates along with the single group III isolate. The single group I isolate is clustered outside of these four groups. The four clusters are significantly different from each other (Kruskal–Wallis tests, *p* > 0.001). It is also worth mentioning that genes for the production of the emetic toxin are not identified in any of the isolates. Genes for the biosynthesis of the anthrax capsule were restricted to isolate N31 (N.B. Hendriksen, Department of Environmental Science, Aarhus University, Denmark. The gene for the protein was detected in the WGS of isolate N31 by Btyper, 2020). 

**Figure 3:**
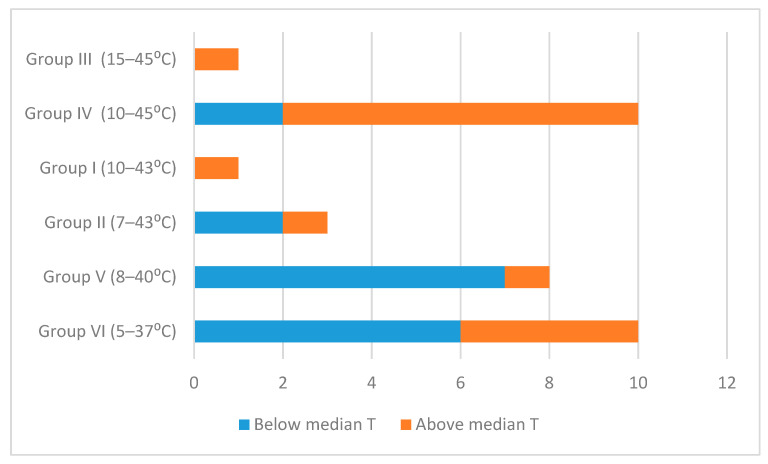
Numbers of 33 *B. cereus sensu lato* isolates in five phylogenetic groups in relation to the annual mean temperature of soils from which they are isolated. Blue refers to numbers of isolates from soils with an annual mean temperature below or equal to the median annual temperature (10.2 °C) of the 17 soils, and brown refers to numbers of isolates from soils with temperature above the median temperature. The temperature growth range of the six phylogenetic groups are shown [2].

Figure 3 shows the distribution of the 33 isolates divided into six phylogenetic groups in relation to the yearly mean temperature of the country where the soils were collected (https://www.weatherbase.com). The distribution is not random, as revealed by Chi-square analysis (*p* < 0.05). This seems to be caused by a higher number of isolates belonging to a phylogenetic group with a growth range above 10 °C present in countries with mean temperatures above the median temperature of all the eleven countries, and then the opposite for the isolates belonging to phylogenetic groups having a growth starting below 10 °C, which are more prevalent in countries with median temperatures below or equal to the median temperature of all the eleven countries.

## 4. Discussion

The genome of 33 *B. cereus* group isolates was whole genome sequenced using Illumina Nextseq. The assembled contigs of the 33 isolates shared comparable genome size and GC-content similar to values previously reported for *B. cereus* group strains [26] The same is the case with the number of tRNA and rRNA genes. The number of contigs varies between 173 and 4682, however the sequencing depth was sufficient to cover the breadth of the genomes. This fragmentation of the assemblies might be caused by the relatively low GC-content, the presence of several extra-chromosomal elements and highly abundant repeat elements [27,28]. In spite of this variation in fragmentation of the assemblies, the predicted number of proteins are similar among the isolates and comparable to numbers previously reported [26], 

The considerable diversity among the 33 isolates, evidenced by the presence of 29 STs, including 11 new ones, most likely reflects that they are soil bacteria that occupy diverse ecological habitats that can survive as spores in soil reservoirs [14,29]. Some of the sequence types are determined with some uncertainty, because no allele in the current database matches with 100% identity to all seven of the analyzed alleles. The high correspondence between the division of the isolates into six phylogenetic groups by *panC*, MLST and ANI analyses is in accordance with the analysis done by Carroll et al. [15] and by Bazinet [6].

The isolates were identified at the species level with four different procedures and resulted in different outputs, as none of the isolates was identified as the same species with all four procedures. The number of species identified by the different procedures varies between 2 and 13. Traditional identification, based on several tests, divided the isolates into the species *B. cereus* and *B. weihenstephanensis*. Recently, it has been demonstrated that *B. weihenstephanensis* is a later synonym for *B. mycoides* [30]. 

Identification based on 16S rRNA gene sequencing divided the isolates into six *B. cereus* group species, while three isolates were not identified to this group. The isolates identified as *B. cereus*, *B. wiedmannii* and *B. toyonensis* are members of more than one of the phylogenetic groups, while isolates belonging to *B. mycoides*, *B. thuringiensis* and *B. pseudomycoides* only belong to one group; however, *B. thuringiensis* and *B. pseudomycoides* are only represented by two or one isolate, respectively. Limitations in the use of 16S rRNA gene sequencing for the phylogenetic-based classification of the *B. cereus* group have previously been shown [31]. The identification of species with Btyper3 is based on WGS and uses a threshold of 92.5% ANI for genomo-species clusters [7]. This methodology divides the isolates into the species *B. mosaicus*, *B. cereus* SS, *B. toyonensis*, *B. mycoides* and *B. pseudomycoides*, and further, four of the isolates belonging to *B. cereus* SS are Biovar *thuringiensis*. This identification reflects the division of the isolates into phylogenetic groups as four of the species correspond to a phylogenetic group, while *B. mosaicus* corresponds to the two neighboring groups II and III. The chosen threshold at 92.5% ANI deviates from the widely accepted 95% ANI threshold for current species definition [32], and it was chosen to create a strong relationship between identification to the species level and the known phylogenetic structure. If a threshold at 95% ANI is chosen for analyzing the 33 isolates, one isolate each of the species *B. mosaicus* and *B. mycoides* must be considered as a new undescribed species according to Figure 1. Further, it must be noted that the Btyper3-based identification means that: (i) some isolates which by traditional methods would be identified as *B. cereus* are now considered as the new species *B. mosaicus*, (ii) isolates which do not have a rhizoid growth on agar plates are identified as *B. mycoides* or *B. pseudomycoides* and (iii) isolates possessing genes such as Cry75Aa3 and different vegetative insecticidal proteins are considered as *Biovar thuringiensis*, although they do not produce crystal proteins, a key identification characteristic for *B. thuringiensis.*

The TYGS-based identification is based on in silico DNA:DNA hybridization and species delimitation as described by Meier-Kolthoff et al. [5]. Based on the procedure, the 33 isolates are divided into the species *B. cereus, B. wiedmanii, B. toyonensis, B. proteolyticus, B. mycoides, B. pseudomycoides* and seven new species. This division of the isolates into species does not correspond to the phylogenetic division of the isolates into six groups. Indeed, although all isolates affiliated to phylogenetic group V are identified as *B. toyonensis*, two isolates also identified as *B. toyonensis* are affiliated to groups II and VI. The three new species comprising more than one isolate affiliated to the species are all restricted to one phylogenetic group, and this might reflect that the *B. cereus* group has a clonal phylogenetic structure [3]. The difference between the ANI-based and the TYGS-based identification reflects that the two methods interpret the phylogeny of the *B. cereus* group differently and use different definitions for the affiliation of isolates to species.

The identification performed by four different procedures points to the importance of describing how identification at the species level for the *B. cereus* group members has been executed.

Most of the 33 isolates across species and phylogenetic groups possessed seventeen of the nineteen analyzed genes for virulence factors, which corresponds to previous results [7,29]. It has also been found previously that *cytK2* and *hlyII* are restricted to phylogenetic groups II, III and IV, and that those genes producing the emetic toxin and the *B. anthracis* capsule are notably restricted to a few clonal clusters in group III [7,15]. We reported a close association between percentage identity of the genes at the protein level to selected standard genes and the distribution of the isolates into six phylogenetic groups. Earlier reports have shown that cytotoxicity varies between the phylogenetic groups, and that *nheA* and *nheB, hblA* and *hblD* and *cytK* genes are polymorphic [8,33,34]. It is unknown for most of these virulence factors whether different variants vary in virulence, except for *CytK*, which exists in two forms, *CytK1* and *CytK2*, that exhibit 89% sequence identity at the protein level. The two *CytK* proteins differ in their biological effects, as *CytK-2* forms pores with a lower conductance than those made by *CytK-1* [34,35,36], and compared to *CytK-2*, *CytK-1* displays a higher toxicity towards human intestinal Caco-2 and Vero cells [35]. This indicates that the variability in the sequences of the different virulence factors can affect their biological effects. This question needs to be investigated further for the other polymorphic virulence factors present in the *B. cereus* group in the future.

Our data also show that distribution of the isolates is related to the mean temperatures of the countries, as the distribution is related to the phylogenetic groups, which in relation to temperature, relates to different ecotypes [2,10,11]. In other words, isolates of the most cold-adapted groups, II, V and VI, are more common in colder soils, while isolates of more mesophylic groups, I, III and IV, prevail in more temperate soils. The relationship between temperature and the geographical distribution of *B. cereus* has been investigated earlier and shown by von Stetten et al. [37].

In conclusion, the phylogeny, identification and characterization of 33 *B. cereus sensu lato* isolates originating from 17 agricultural soils from 11 countries were analyzed on the basis of whole genome sequencing. Phylogenetic analyses divided the isolates into six groups that follow the phylogenetical division of *B. cereus sensu lato* proposed by several authors [1,2,3,6,7]. Identifications were based on four different methods with varied results, as none of the isolates were identified as the same species by all four methods: only the recent identification method proposed by Carroll et al. [7] directly reflected the phylogeny of the isolates. This points to the importance of describing the basis and method used for the identification. The presence and percent identity of the protein product of 19 genes potentially involved in pathogenicity divided the 33 isolates into four statistically different clusters, which corresponds to the generally accepted phylogenetic groups II, IV, V and VI. Phylogenetic groups I and III were each only presented by one isolate. This shows that different pathotypes exist and that it is possible to differentiate between them by comparing the percent identity of proteins potentially involved in pathogenicity. It also shows that a basic link between phylogeny and pathogenicity exists. Finally, the geographical distribution of the isolates was not random: they were distributed in relation to their division into the six phylogenetic groups, which again relates to different ecotypes with different temperature growth ranges [2,10,11]. This means that we find it easier to analyze and understand the results obtained about the 33 *B. cereus sensu lato* isolates in a phylogenetic, patho-type and ecotype-oriented context, than in a context based on a method-dependent identification at the species level.

## Figures and Tables

**Table 1:** Origin and characteristics of agricultural fields used for isolation of *B. cereus sensu lato* bacteria.

Isolate Number	Country	GPS-Coordinates	Meters above Sea Level	Crop	Yearly Mean Temperature (°C)
N1 and N2	Lithuania	54.864 23.941	50	Vegetables	6.4
N3 and N4	Tunisia	34.5117 10.4924	200	Olive trees	18.1
N5 and N6	Netherlands	51.966 5.655	9	Leak	9.2
N7 and N8	Slovenia	46.1434 14.5580	265	Oat	10.4
N9 and N10	Switzerland	46.3919 6.21164	450	Livestock	9.2
N11 and N12	Hungary	47.3827 17.4992	135	Maize	11.1
N13 and N14	Hungary	46.0559 19.2622	95	Pea	11.1
N15 and N16	Serbia	45.1908 19.2101	88	Fallow	11.3
N17 and N18	Serbia	45.1908 19.2101	88	Fallow	11.3
N19 and N20	Turkey	39.9672 32.6623	938	Lemon	10.9
N21 and N22	Turkey	40.0808 34.1200	780	Wheat, Barley	10.9
N23 and N24	Turkey	39.9040 32.6345	900	Garden	10.9
N25 and N26	Turkey	39.5456 33.4436	1000	Grass	10.9
N27 and N28	Turkey	40.0047 32.5142	1066	Grass	10.9
N29 and N30	Denmark	55.697 12.103	5	Grass	7.8
N31 and N32	Germany	52.359 13.308	42	Potato	10.2
N33 and N34	Belgium	51.138 3.938	6	Tree Nursery	10.2

**Table 2:** Overall whole genome characteristics of 33 *B. cereus sensu lato* isolates. Bases = number of nucleotides, Contigs = number of contigs, CDS = number of coding sequences, tRNA = number of tRNA genes, rRNA = number of rRNA genes; GC-content = percentage guanine-cytosine content

Isolate	Bases	Contigs	CDS	tRNA	rRNA	GC content
N1	6589056	1486	6332	139	12	34.87
N2	6208593	362	6112	116	10	34.7
N3	5981260	1394	5801	98	10	35.18
N4	5628374	283	5577	115	11	35.01
N5	6039339	435	6023	120	9	35.08
N6	6050091	858	6102	139	12	35.33
N7	7090662	4682	6057	129	14	37.86
N8	5963382	392	6054	102	12	35.2
N9	5731016	563	5730	112	12	35.31
N10	5648760	410	5647	128	11	35.25
N11	6015328	286	5869	109	12	35.06
N12	6179004	1900	5902	125	16	35.4
N13	5920438	304	5853	112	13	34.9
N14	5687870	332	5733	132	8	35.17
N15	6040251	455	5965	126	12	34.99
N16	5783502	405	5769	120	11	35.09
N17	6225349	391	6198	98	7	34.91
N18	5976955	349	5862	94	10	35.05
N19	6310391	1305	6317	105	9	35.3
N20	6366475	989	6433	104	8	35.18
N21	6047278	805	6041	112	20	35.48
N22	5686548	425	5689	122	11	35.1
N23	6246356	279	6250	105	7	34.76
N24	6083667	332	6065	100	7	34.92
N25	6552745	545	6375	96	6	34.68
N26	6346459	979	6410	101	7	35.18
N27	6484007	279	6360	91	6	34.67
N28	5996583	320	6003	102	10	35,13
N29	5603679	484	5593	95	11	35.18
N30	5706629	631	5665	99	9	35.3
N31	5996429	274	5885	94	11	34.98
N33	5528708	290	5550	109	14	35.32
N34	5640513	173	5616	92	9	35.28

**Table 3:** *PanC* and multiple loci sequence typing (MLST) characterization of 33 *B. cereus sensu lato* isolates. * Determined with uncertainty: ? = New profile. *glp* (glycerol uptake facilitator protein), *gmk* (guanylate kinase, putative), *ilvD* (dihydroxy-acid dehydratase), *pta* (phosphate acetyltransferase), *pur* (phosphoribosylaminoimidazolecarboxamide), *pycA* (pyruvate carboxylase), *tpi* (triosephosphate isomerase).

NR	Country	Phylogenetic Group (panC)	MLST Profile	*glp*	*gmk*	*ilv*	*pta*	*pur*	*pyc*	*tpi*
N1	Lithuania	clade 4	?	14	8 *	48	45	58	51	7
N2	Lithuania	clade 4	?	285	8	283 *	45 *	58	87	7
N3	Tunesia	clade 5	223	43	26	35	42	39	41	63
N4	Tunesia	clade 4	1009	16	6	170	9	4	7	21
N5	Nederlands	clade 2	616	81	53	117	71	113	93	80
N6	Nederlands	clade 2	? *	110	56	111*	188	64	96	26
N7	Slovenia	clade 6	434	26	21	126	104	78	32	18
N8	Slovenia	clade 6	? *	274	115	227	200	195	170	163 *
N9	Switzerland	clade 6	428	108	51	130	121	109	92	79
N10	Switzerland	clade 6	434	26	21	126	104	78	32	18
N11	Hungary	clade 5	? *	87	26 *	78	90	273	41	30
N12	Hungary	clade 5	?	87	26	78	90	273	220	30
N13	Hungary	clade 4	23	15	7	7	2	5	8	13
N14	Hungary	clade 2	? *	118 *	35	171 *	22	96	20	26
N15	Serbia	clade 5	72 *	43	26 *	35	40	39	41	30
N16	Serbia	clade 3	1766 *	229 *	5	271	240	281	183	190
N17	Serbia	clade 5	278	83	26	35	42	39	71	30
N18	Serbia	clade 5	1484 *	43	26	78	42	39 *	71	30 *
N19	Turkey	clade 6	? *	75 *	104 *	303 *	15 *	103 *	202 *	128 *
N20	Turkey	clade 4	56	15	7	7	2	7	26	13
N21	Turkey	clade 1	? *	134	13	274	253	84	44	35 *
N22	Turkey	clade 4	4	13	8	8	11	11	12	7
N23	Turkey	clade 4	12	15	7	7	2	7	10	13
N24	Turkey	clade 5	886	43	50	35	125	70	41	30
N25	Turkey	clade 4	12	15	7	7	2	7	10	13
N26	Turkey	clade 4	56	15	7	7	2	7	26	13
N27	Turkey	clade 4	12	15	7	7	2	7	10	13
N28	Turkey	clade 6	?	8	10	22	15	35	9	11
N29	Denmark	clade 6	635 *	26	21	104 *	68	27*	32	18
N30	Denmark	clade 6	? *	108	139	104	15 *	221	92	79
N31	Austria	clade 5	487	83	26	143	133	91	41	30
N33	Belgium	clade 6	254	103	63	5	15	94	70	11
N34	Belgium	clade 6	734	25	10	22	165	83	23	11

**Table 4:** Identification of 33 *B. cereus sensu lato* by four different methods.

Isolate	Country	Phylogeny	Traditional	16S RNA gene	Species ANI	Species DNA/DNA
N21	Turkey	clade 1	*B. cereus*	*B. cereus*	*B. pseudomycoides*	*B pseudomycoides*
N14	Hungary	clade 2	*B. cereus*	*B. cereus*	*B. mosaicus*	*B toyonensis*
N16	Serbia	clade 2	*B. cereus*	*B. cereus*	*B. mosaicus*	New species 2
N5	Nederlands	clade 2	*B. cereus*	*B. cereus*	*B. mosaicus*	New species 3
N6	Nederlands	clade 2	*B. cereus*	*B. cereus*	*B. mosaicus*	New species 5
N1	Lithuania	clade 4	*B. cereus*	*B. cereus*	*B. cereus s.s.* *biovar Thuringiensis*; *B. thuringiensis*	New species 1
N13	Hungary	clade 4	*B. cereus*	*B. cereus*	*B. cereus s.s.*	*B. wiedmanii*
N2	Lithuania	clade 4	*B. cereus*	*B. cereus*	*B. cereus s.s.* *biovar Thuringiensis*; *B. thuringiensis*	New species 1
N20	Turkey	clade 4	*B. cereus*	*B. cereus*	*B. cereus s.s.*	New species 7
N22	Turkey	clade 4	*B. cereus*	*B. cereus*	*B. cereus s.s.*	*B cereus*
N23	Turkey	clade 4	*B. cereus*	*B. mycoides*	*B. cereus s.s.*	New species 7
N25	Turkey	clade 4	*B. cereus*	*B. mycoides*	*B. cereus s.s.* *biovar Thuringiensis*; *B. thuringiensis*	New species 7
N26	Turkey	clade 4	*B. cereus*	*B. mycoides*	*B. cereus s.s.*	New species 7
N27	Turkey	clade 4	*B. cereus*	*B. mycoides*	*B. cereus s.s.* *biovar Thuringiensis*; *B. thuringiensis*	New species 7
N4	Tunesia	clade 4	*B. cereus*	*B. mycoides*	*B. cereus s.s.*	*Bacillus cereus*
N11	Hungary	clade 5	*B. cereus*	*B. mycoides*	*B. toyonensis*	*B toyonensis*
N12	Hungary	clade 5	*B. cereus*	*B. mycoides*	*B. toyonensis*	*B. toyonensis*
N15	Serbia	clade 5	*B. cereus*	*B. mycoides*	*B. toyonensis*	*B.toyonensis*
N17	Serbia	clade 5	*B. cereus*	*B. pseudomycoides*	*B. toyonensis*	*B toyonensis*
N18	Serbia	clade 5	*B. cereus*	*B. sp*	*B. toyonensis*	*B toyonensis*
N24	Turkey	clade 5	*B. cereus*	*B. sp.*	*B. toyonensis*	*B toyonensis*
N3	Tunesia	clade 5	*B. cereus*	*B. thuringiensis*	*B. toyonensis*	*B. toyonensis*
N31	Austria	clade 5	*B. cereus*	*B. thuringiensis*	*B. toyonensis*	*B toyonensis*
N10	Switzerland	clade 6	*B. weihenstephanensis*	*B. toyonensis*	*B. mycoides*	*B toyonensis*
N19	Turkey	clade 6	*B. weihenstephanensis*	*B. wiedmanii*	*B. mycoides*	New species 6
N28	Turkey	clade 6	*B. weihenstephanensis*	*B. wiedmanii*	*B. mycoides*	*B. mycoides*
N29	Denmark	clade 6	*B. weihenstephanensis*	*B. wiedmanii*	*B. mycoides*	New species 4
N30	Denmark	clade 6	*B. weihenstephanensis*	*B. wiedmanii*	*B. mycoides*	New species 4
N33	Belgium	clade 6	*B. weihenstephanensis*	*B. wiedmanii*	*B. mycoides*	*B mycoides*
N34	Belgium	clade 6	*B. weihenstephanensis*	*B. wiedmanii*	*B. mycoides*	*B mycoides*
N7	Slovenia	clade 6	*B. weihenstephanensis*	*B. wiedmanii*	*B. mycoides*	*B. proteolyticus*
N8	Slovenia	clade 6	*B. weihenstephanensis*	Not identified	*B. mycoides*	*Bacillus proteolyticus*
N9	Switzerland	clade 6	*B. weihenstephanensis*	*B. mycoides*	*B. mycoides*	New species 4

**Table 5:** Presence and percentage identity to the translated genes found in the BTyper virulence database of 19 genes potentially involved in pathogenesis at the protein level in 33 *B. cereus sensu lato* isolates.

Isolate	Group	cerA	cerB	clo	cytK2	entA	entFM	hblA	hblB	hblC	hblcD	hlyII	inhA1	inhA2	nheA	nheB	nheC	plcA	plcB	sph
N21	I	89	0	0	0	91	71	89	0	85	88	0	83	86	74	86	79	0	89	0
N14	II	95	90	98	0	96	94	95	87	94	99	0	94	96	96	100	95	94	95	98
N16	II	95	88	96	0	94	92	99	96	99	100	96	94	96	97	100	94	96	95	96
N5	II	96	89	99	0	94	95	95	86	94	100	96	93	97	97	100	95	94	96	97
N6	II	95	90	95	0	97	95	98	87	95	99	95	94	96	97	99	95	94	95	98
N1	IV	100	94	99	98	98	97	99	98	98	100	0	98	99	99	100	99	95	100	91
N13	IV	100	95	99	0	95	95	99	98	98	100	99	94	99	100	100	98	96	100	91
N2	IV	100	94	99	99	97	92	99	98	98	100	0	98	99	99	100	98	95	100	90
N20	IV	100	98	99	0	95	95	99	98	98	100	99	94	99	100	100	98	96	100	90
N22	IV	100	95	99	100	99	97	99	99	100	100	100	100	100	100	100	99	100	100	92
N23	IV	100	95	99	0	95	93	99	98	98	100	99	94	99	100	100	98	96	100	91
N25	IV	100	95	99	97	95	94	99	98	98	100	99	94	98	100	100	98	96	100	91
N26	IV	100	0	99	0	95	95	99	98	98	100	99	94	99	100	100	98	96	100	90
N27	IV	100	95	99	97	95	94	99	98	98	100	99	94	98	100	100	98	96	100	91
N4	IV	100	95	99	99	98	95	99	98	99	100	99	94	99	99	100	98	97	100	92
N11	V	98	90	91	0	97	90	93	86	94	99	0	96	97	97	100	93	90	98	94
N12	V	99	89	92	0	95	90	92	86	94	99	0	96	97	97	100	94	90	99	93
N15	V	98	89	93	0	96	90	95	85	95	99	0	94	97	96	100	94	90	98	93
N17	V	99	89	0	0	97	90	95	86	95	100	0	94	97	96	100	94	90	99	93
N18	V	99	89	0	0	97	90	95	86	95	100	0	94	97	96	100	94	90	99	93
N24	V	99	89	93	0	97	90	95	86	95	99	0	94	97	96	100	94	91	99	93
N3	V	98	89	93	0	96	90	95	86	95	99	0	94	97	96	100	94	90	98	93
N31	V	99	89	93	98	97	90	95	86	94	99	0	95	97	97	100	93	91	99	93
N10	VI	98	89	97	0	88	88	92	87	84	94	0	91	93	94	99	93	93	98	92
N19	VI	98	87	89	0	88	90	69	65	79	0	0	91	94	96	100	94	95	98	92
N28	VI	95	87	97	0	88	87	97	88	93	98	0	91	95	97	100	92	0	95	90
N29	VI	97	88	0	0	88	89	91	87	84	94	0	93	96	95	100	93	92	97	91
N30	VI	98	89	98	0	88	88	90	87	88	96	0	91	94	96	100	93	93	98	92
N33	VI	95	87	98	0	94	88	91	89	89	93	0	91	93	97	100	93	93	95	91
N34	VI	95	87	95	0	93	88	98	87	93	99	0	91	95	95	98	94	93	95	91
N7	VI	98	89	97	0	88	88	92	87	84	94	0	91	93	94	99	93	93	98	92
N8	VI	91	88	98	0	88	85	91	87	84	92	0	91	92	95	99	94	0	91	89
N9	VI	98	87	98	0	87	89	90	89	88	96	0	91	93	96	99	93	92	98	92
